# A Soy-Derived Isoflavone Genistein Inhibits the Life Cycle of PRRSV In Vitro

**DOI:** 10.3390/ijms27188235

**Published:** 2026-09-16

**Authors:** Dan Cao, Changchao Huan, Xiaofei Tang, Yongguo Xue

**Affiliations:** 1 Soybean Research Institute, Heilongjiang Academy of Agricultural Sciences, Harbin 150086, China; caodan0825@163.com (D.C.); xftang@126.com (X.T.); 2 Institute of Agricultural Science and Technology Development, College of Veterinary Medicine, Yangzhou University, Yangzhou 225009, China; changchaohuan@yzu.edu.cn; 3 Jiangsu Co-Innovation Center for Prevention and Control of Important Animal Infectious Diseases and Zoonoses, Yangzhou 225009, China; 4 Key Laboratory of Avian Bioproduct Development, Ministry of Agriculture and Rural Affairs, Yangzhou 225009, China

**Keywords:** Soybean, genistein, PRRSV, antiviral mechanism, autophagy

## Abstract

Porcine reproductive and respiratory syndrome virus (PRRSV) has become a global health threat for swine. Because of the limited effectiveness of commercial vaccine strategies and the absence of specific antiviral chemical drugs for PRRS, it is urgent to identify new strategies for preventing and controlling PRRSV infections. Genistein, a soy-derived isoflavone, has shown promise in reducing viremia in PRRSV-infected pigs. However, its antiviral molecular mechanism remains unclear. This study investigates the inhibitory effects and mechanism of genistein on PRRSV infection in vitro. Our results demonstrated that genistein dose-dependently inhibited PRRSV infection in Marc-145 cells at several stages of the PRRSV replication cycle. With its favorable safety profile and potent efficacy in antiviral and growth promotion in swine, genistein stands out as a promising dietary adjunct for PRRSV prevention and control in swine husbandry.

## 1. Introduction

Porcine reproductive and respiratory syndrome (PRRS), caused by Porcine reproductive and respiratory syndrome virus (PRRSV), continues to be one of the most economically devastating swine diseases worldwide [1]. PRRS is characterized by reproductive failure in sows and respiratory disease in piglets with high mortality [2,3,4]. PRRSV is an enveloped, positive-sense, single-stranded RNA virus which belongs to the family *Arteriviridae* and order *Nidovirales*. PRRSV is divided into two different species, PRRSV-1 and PRRSV-2, which exhibit only 60% nucleotide identity and provide only partial cross protection to each other [5]. Viral genetic diversity and variation with recombination and immunosuppression complicates vaccine development and disease control [6]. Despite extensive research over three decades, effective antiviral strategies remain limited, and current management relies primarily on modified live vaccines and biosecurity measures. Consequently, there is an urgent need to develop effective antiviral agents to prevent and control PRRSV infection [7].

Genistein, a naturally occurring isoflavone derived primarily found in soybeans, exhibits diverse biological activities, including estrogenic like activity, antioxidant, anti-inflammatory, and tyrosine kinase inhibitory properties [8]. This phytoestrogen has attracted considerable attention as a potential antiviral and immune-modulatory agent [9,10,11]. It was reported that genistein was effective against several viruses, including human immunodeficiency virus, herpes simplex virus type 1, herpes B virus, and rotavirus infection [12,13,14,15,16]. It also exhibits notable antiviral efficacy against animal viruses responsible for severe livestock and poultry diseases, such as avian leucosis virus, bovine herpes virus 1, and African swine fever virus [17,18,19]. Previous studies have demonstrated that dietary supplementation with genistein at 200–800 mg/kg (ppm) reduced PRRSV viremia and improved average daily gain in experimentally challenged pigs, suggesting its potential as a feed additive for controlling PRRSV infection [20,21]. However, the precise molecular mechanisms underlying its antiviral activity have remained uncovered.

The present study was designed to systematically investigate the antiviral mechanism of genistein against PRRSV in vitro. Our results demonstrate that genistein exerts potent inhibitory effects on PRRSV infection in a dose-dependent manner, with significant suppression observed at concentrations ranging from 20 to 80 μM without inducing significant cytotoxicity. Collectively, we demonstrate that genistein inhibits PRRSV infection primarily by blocking viral attachment, entry, and replication without affecting later stages of the viral life cycle such as assembly and release.

## 2. Results

### 2.1. Toxicity of Genistein Toward Marc-145 Cells and Primary PAMs

**Figure 1:**
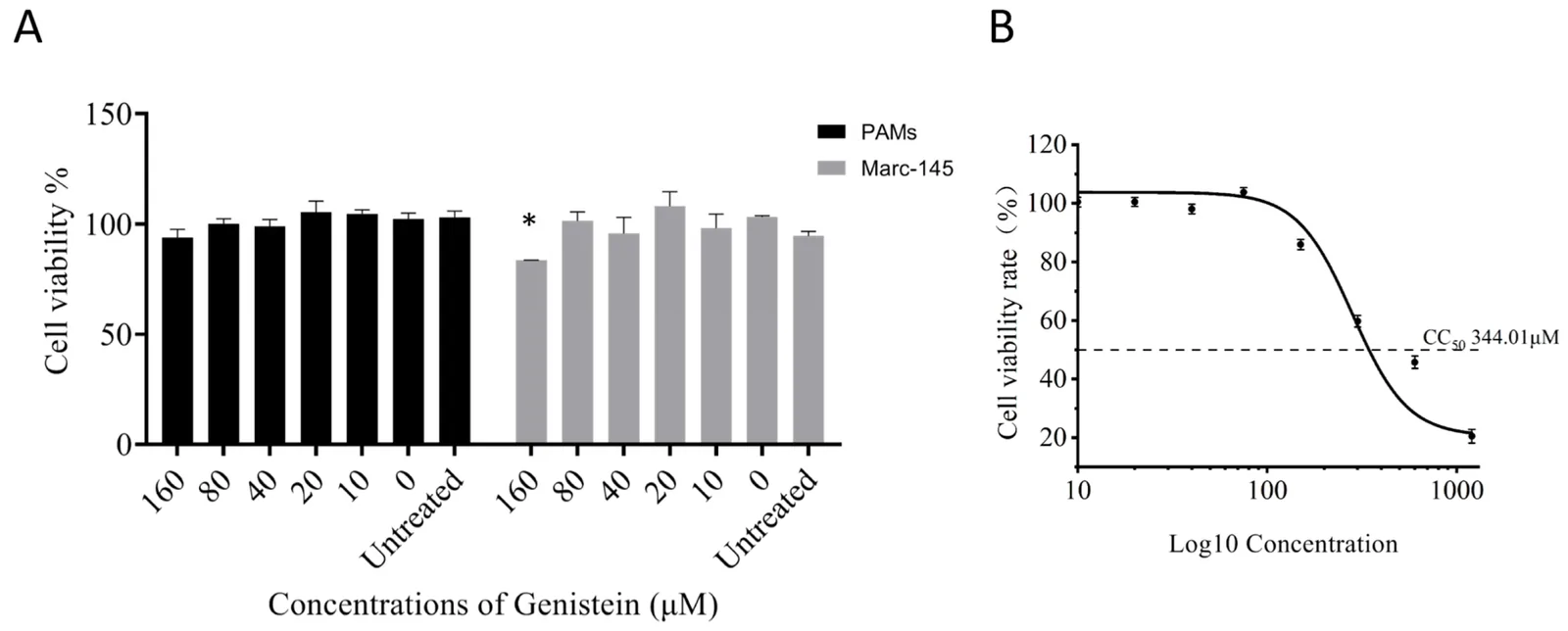
(**A**) The cytotoxicity of genistein on Marc-145 cells and PAMs were determined using the CCK-8 assay kit. (**B**) The 50% cytotoxic concentration (CC_50_) on Marc-145 cells was subsequently calculated using a non-linear regression analysis. Dates are presented as the mean of three independent experiments. *, *p* < 0.05.

The cytotoxic effect of Genistein on Marc-145 cells and primary PAMs were measured using the CCK-8 assay after 48 h co-incubation. Results revealed genistein concentrations ranging from 10 to 160 μM without inducing significant cytotoxicity in PAMs, while Marc-145 cell proliferation was inhibited by genistein at concentrations above 160 μM (Figure 1A). The CC_50_ value of genistein on Marc-145 cells was calculated 344.01 μM (Figure 1B). Therefore, genistein concentration below 80 μM was selected for the following experiments.

### 2.2. Antiviral Effect of Genistein on PRRSV

**Figure 2:**
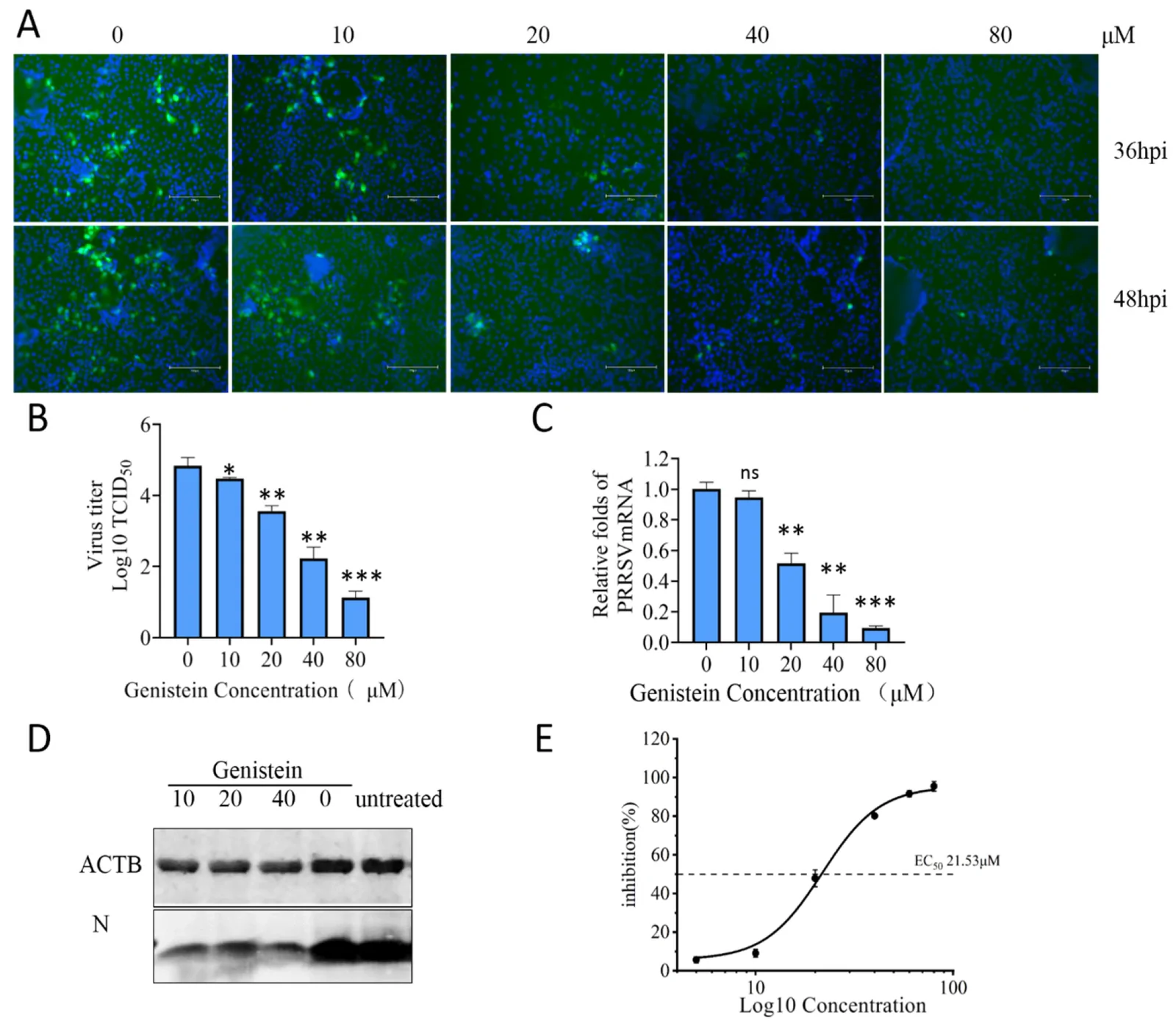
Inhibitory effect of genistein on PRRSV replication in viral titers, viral RNA, and protein. (**A**) The fluorescence of PRRSV-GFP in Marc-145 cells treated with genistein at concentrations of 0–80 μM was monitored. GFP expression served as an indicator of viral infection, while DAPI was used for nuclear staining. Scale bar, 150 μm. (**B**) The virus titer was determined by TCID_50_. (**C**) Intracellular viral RNA copy numbers were quantified by RT-qPCR at 24 h post infection. (**D**) The expression of PRRSV N protein expression was tested by Western blotting with antibodies against N protein. (**E**) The half-maximal effective concentration (EC_50_) was calculated with inhabitation of RNA copies according RT-qPCR. *, *p* < 0.05; **, *p* < 0.01; ***, *p* < 0.001; ns, not significant.

To investigate the antiviral effect of genistein against PRRSV in vitro, Marc-145 cells were infected with PRRSV-GFP (0.1 MOI) with genistein at different concentrations (0 μM, 20 μM, 40 μM, and 80 μM) for 36 and 48 h, and the fluorescent intensity of GFP in infected cells was observed. Compared with the untreated group, genistein treatment reduced the GFP fluorescence signal significantly, especially at the concentrations of 20 μM with an inhabitation rate above 80%, suggesting genistein inhibited the replication of PRRSV-GFP. The antiviral effect of genistein was further demonstrated using wild PRRSV through TCID_50_ assay, RT-qPCR, and Western blot. Marc-145 cells were infected with PRRSV at 0.1 MOI with genistein for 36 h, cell supernatant was collected to determine the viral titer in terms of TCID_50_, and the results indicated that genistein treatment reduced the production of PRRSV virions (Figure 2B). RT-qPCR analysis indicated that genistein decreased the viral RNA copies, 80.16% at 40 μM and 95.5% at 80 μM (Figure 2C). The WB result showed the expression level of N protein was reduced after genistein treatment (Figure 2D). The concentration for 50% of maximal effect (EC_50_) value was calculated to be 21.53 μM. The selectivity index (SI) of genistein was calculated as the ratio of CC_50_ to EC_50_. With a CC_50_ of 344.01 μM and an EC_50_ of 21.53 μM, the SI was determined to be approximately 16. These results indicate that genistein treatment inhibited the replication of PRRSV in Marc-145 cells in a dose-dependent manner.

### 2.3. Effects of Genistein on Different Stages of the PRRSV Replication Cycle

**Figure 3:**
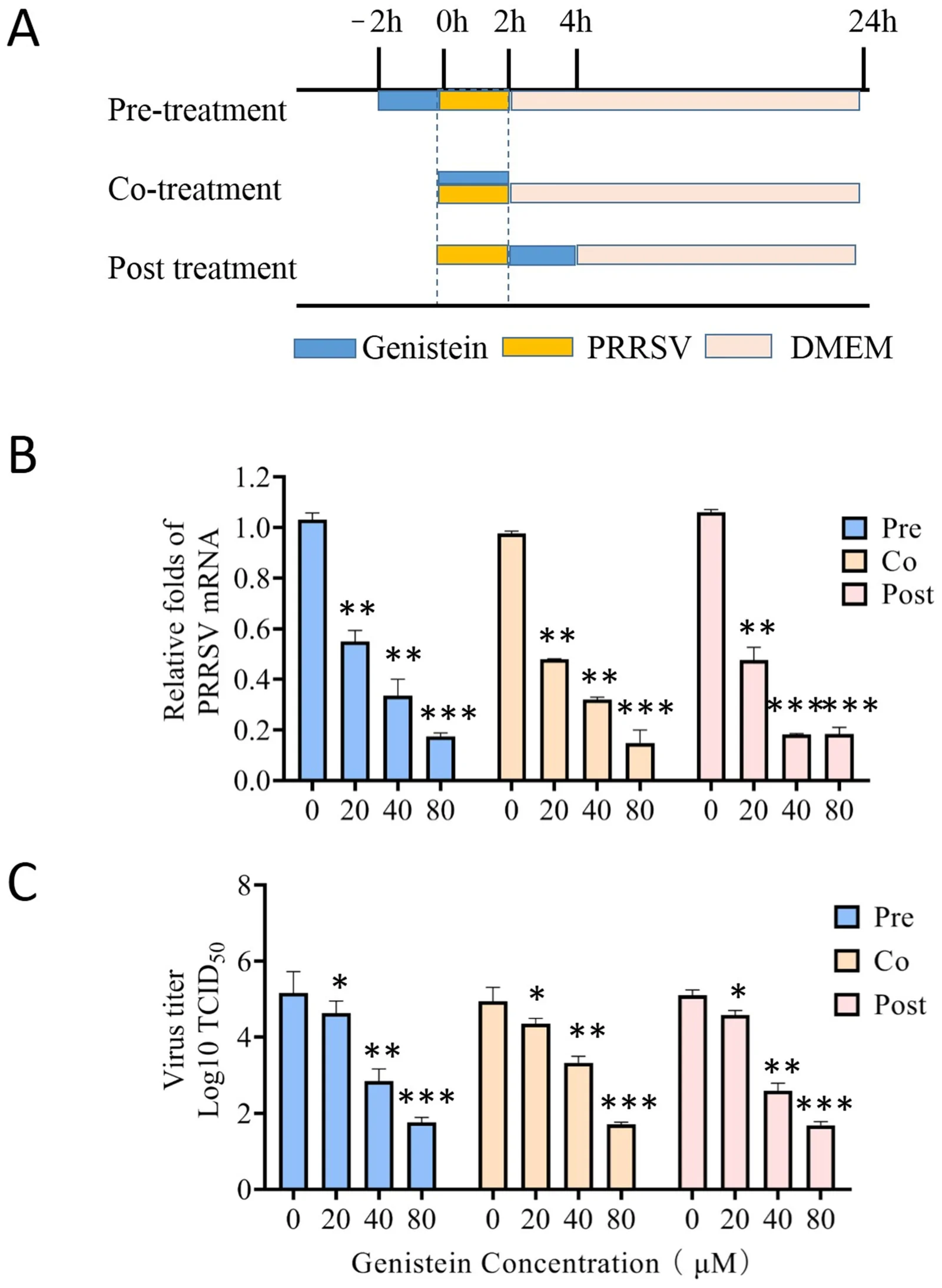
Genistein exerts antiviral activity at multiple stages of PRRSV replication cycle. (**A**) Schematic illustration of time-of-addition assay. Cells were inoculated with PRRSV, and exposed to genistein before, during, or after infection according the schedule. Cells and supernatants were collected at 24 hpi for subsequent analysis. (**B**) PRRSV RNA relative expression analyzed by RT-qPCR. (**C**) Virus titers tested by TCID_50_. Each experiment was performed with three replicates. *, *p* < 0.05; **, *p* < 0.01; ***, *p* < 0.001, compared with the 0 μM genistein treatment group.

In order to explore the specific stage(s) of PRRSV life cycle targeted by genistein, a time-of-addition assay was performed. Marc-145 cells were subjected to genistein (0, 20, 40, 80 μM) via pre-treatment, co-treatment, or post-treatment regimen during PRRSV infection (Figure 3A). Quantitative analysis of viral RNA revealed a significant reduction in copy number across all three treatment modalities, with the inhibitory effect exhibiting a dose-dependent relationship (Figure 3B). Consistent with these results, viral titers were markedly and dose-dependently diminished following pre-treatment, co-treatment, and post-treatment with genistein (Figure 3C). Collectively, these results suggest that genistein suppresses PRRSV replication in Marc-145 cells prior to, during, and subsequent to viral infection.

### 2.4. Impact of Genistein on PRRSV Adsorption, Entry, and Replication

**Figure 4:**
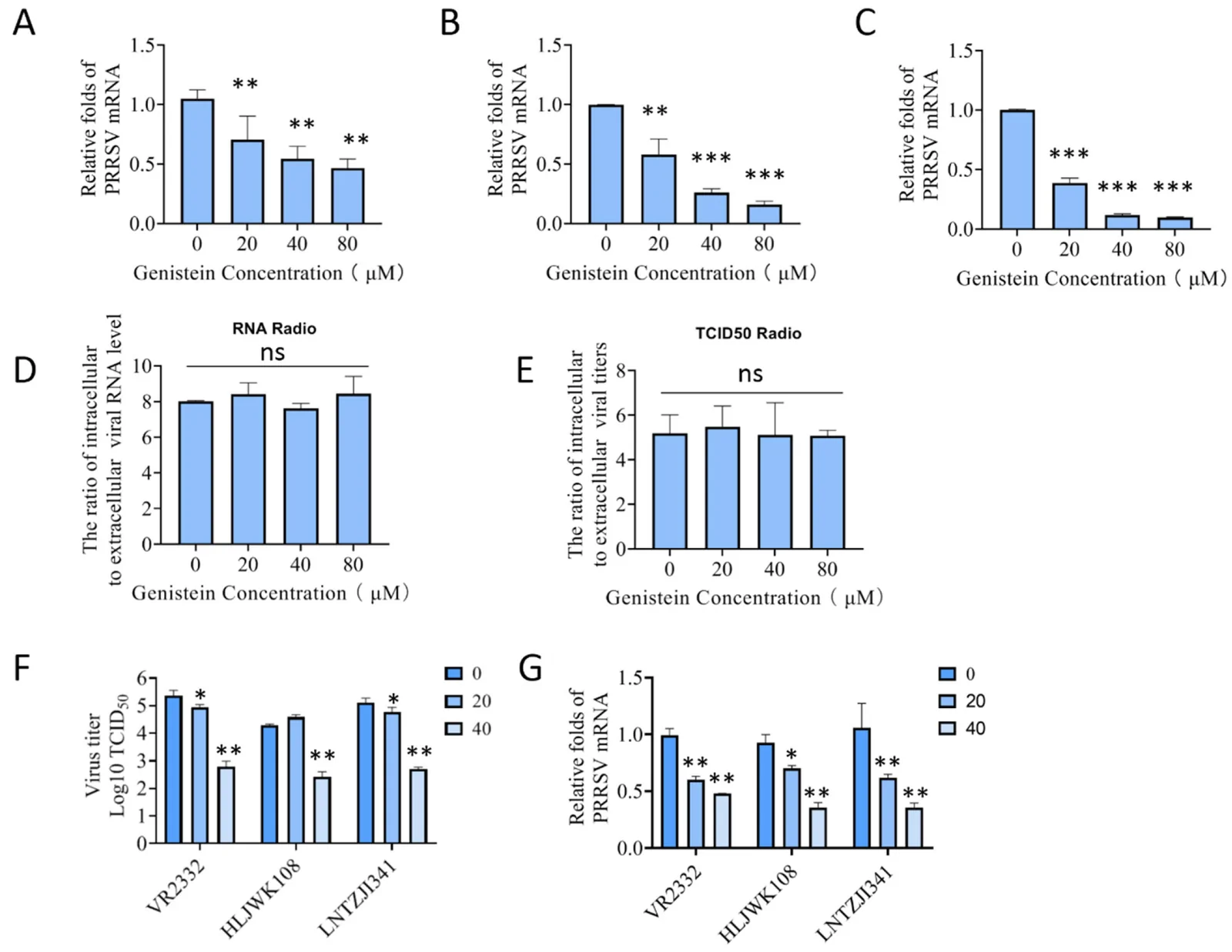
Effects of genistein on PRRSV adsorption, entry, and replication. (**A**) To assess the impact on viral adsorption, Marc-145 cells were infected with PRRSV at 4 °C for 1 h in the presence or absence of genistein. RNA level was quantified by RT-qPCR at 24 hpi. (**B**) For the entry assay, Marc-145 cells were incubated with PRRSV at 4 °C for 1 h, then shifted to 37 °C with genistein treatment. Following citric acid wash to remove surface-bound virions, viral RNA was measured by RT-qPCR at 24 hpi. (**C**) Marc-145 cells were incubated with PRRSV at 37 °C for 1 h, after which genistein was added and maintained at 37 °C. RNA was quantified by RT-qPCR at 6 hpi. (**D**) Marc-145 cells were infected with PRRSV for 20 h, then genistein was added for another 6 h. The cells and supernatants were collected to quantify the viral RNA and virus titer, respectively. The ratio of extracellular to intracellular PRRSV RNA copies and (**E**) the ratio of extracellular to intracellular PRRSV titers was determined. Marc-145 cells were infected with different PRRSV strains for 20 h, genistein with different concentrations (0 μM, 20 μM, 40 μM, and 80 μM) was added for another 6 h. Virus titer (**F**) and viral RNA (**G**) were tested. Each experiment was performed with three replicates. The data are expressed as mean ± SD. *, *p* < 0.05; **, *p* < 0.01; ***, *p* < 0.001, compared with 0 μM genistein treatment group.

Adsorption is the first step of the infection stage; Marc-145 cells were incubated with PRRSV for 1 h at 4 °C with different concentrations (0 μM, 20 μM, 40 μM, and 80 μM) of genistein, the infected cells were washed with cold PBS and cultured at 37 °C for 24 h, and viral RNA copies of adsorption were analyzed by RT-qPCR after 1 h. Figure 4A reveals that the genistein treatment reduced PRRV RNA copies compared with that in control cells; this result indicated that genistein attenuated PRRSV adsorption to Marc-145 cells.

It was reported that PRRSV entry into Marc-145 cells is dependent on the EGFR-PI3K-AKT signaling axis [22]. To investigate whether PRRSV entry was affected by genistein, cells were incubated with PRRSV at 4 °C for 1 h to permit viral adsorption, followed by incubation with genistein at 37 °C to facilitate viral entry. At the end of the incubation, cells were washed with citric acid. Then the viral copy number were quantified by RT-qPCR. The result showed that genistein reduced viral RNA significantly compared with that in the control group (Figure 4B).

In the viral replication assay, the cells were incubated with PRRSV at 37 °C to allow adsorption and entry, after which infected cells were maintained in the presence of genistein at 37 °C. At 6 hpi, cells were harvested for quantification of the PRRSV RNA level. RT-qPCR analysis revealed that genistein decreased viral RNA replication compared with the untreated control group, (Figure 4C). Interestingly, genistein at equivalent concentrations exhibited the most pronounced antiviral effect during the replication stage, followed by the entry stage, with comparatively weaker inhibition observed during the adsorption stage. These findings indicate that the antiviral activity of genistein is primarily exerted during the entry and replication phases of the PRRSV life cycle.

### 2.5. Analysis of PRRSV Assembly and Release Following Genistein Treatment

To test whether genistein influences viral assembly and release, Marc-145 cells were infected with PRRSV for 20 h. Then, genistein was added for another 6 h. The cells and supernatants were respectively collected to quantify the viral RNA by RT-qPCR and to test virus titer by TCID_50_ assay. The ratio of extracellular to intracellular PRRSV RNA copies suggests that genistein had no effect on viral assembly (Figure 4D). Simultaneously, the ratio of extracellular to intracellular PRRSV titers was determined, there was no significant difference among genistein treatments and untreated groups, suggesting that genistein had no effect on PRRSV release (Figure 4E).

### 2.6. Antiviral Activity of Genistein in PAMs and the Antiviral Activity Against Diverse PRRSV Strains

In order to determine whether the antiviral efficacy against PRRSV with genistein was broad-spectrum, genistein was added in cells infected with several PRRSV strains. At 24 hpi, viral RNA and PRRSV virus titers were performed by RT-qPCR and TCID_50_ assay. As shown in Figure 4F,G, genistein reduced both viral RNA and virus titers. These results suggest that genistein at 40 μM exerts noticeable antiviral effects against various viral strains.

**Figure 5:**
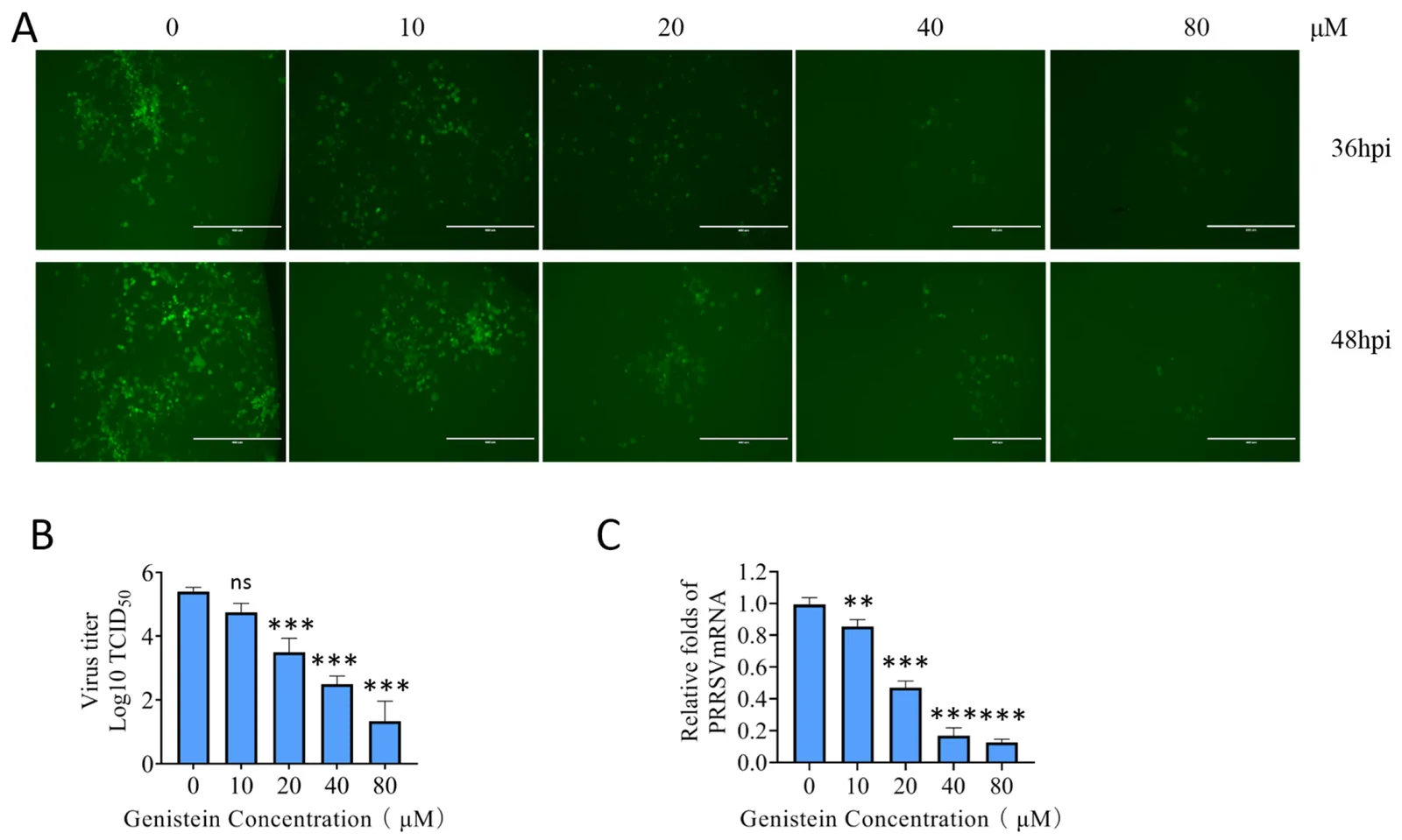
Inhibitory effect of genistein on PRRSV replication in PAMs. (**A**) The fluorescence of PRRSV-GFP in PAMs treated with genistein at concentrations of 0, 10, 20, 40, and 80 μM was monitored. GFP expression served as an indicator of viral infection. Scale bar, 400 μm. (**B**) The virus titer was determined by TCID50. (**C**) Intracellular viral RNA copy numbers were quantified by RT-qPCR. **, *p* < 0.01; ***, *p* < 0.001, compared with 0 μM genistein treatment group.

To determine the antiviral efficacy against PRRSV with genistein in primary porcine alveolar macrophages (PAMs), the natural target cells in vivo, we performed antiviral experiments in PAMs. Primary PAMs were isolated from the lung lavage fluid of 28-day-old healthy specific pathogen-free (SPF) piglets. PAMs were infected with PRRSV-GFP (0.1 MOI) with genistein at different concentrations (0 μM, 20 μM, 40 μM, and 80 μM) for 36 and 48 h, then the fluorescent intensity of GFP was observed. Genistein treatment at concentrations of 10–80 μM reduced the GFP fluorescence signal, suggesting genistein inhibited the replication of PRRSV-GFP in PAMs (Figure 5A). The antiviral effect of genistein was also demonstrated using wild PRRSV through TCID50 assay and RT-qPCR. PAMs were infected with PRRSV with genistein for 36 h, cell supernatant was collected to determine the viral titer, and the results indicated that genistein reduced the virus in cell supernatant (Figure 5B). RNA extraction and RT-qPCR were performed on PAMs; the results indicated that genistein at concentrations of 10 μM, 20 μM, 40 μM, and 80 μM significantly decreased the viral RNA copies compared with 0 μM (Figure 5C).

### 2.7. Modulation of Autophagy by Genistein in PRRSV-Infected Cells

**Figure 6:**
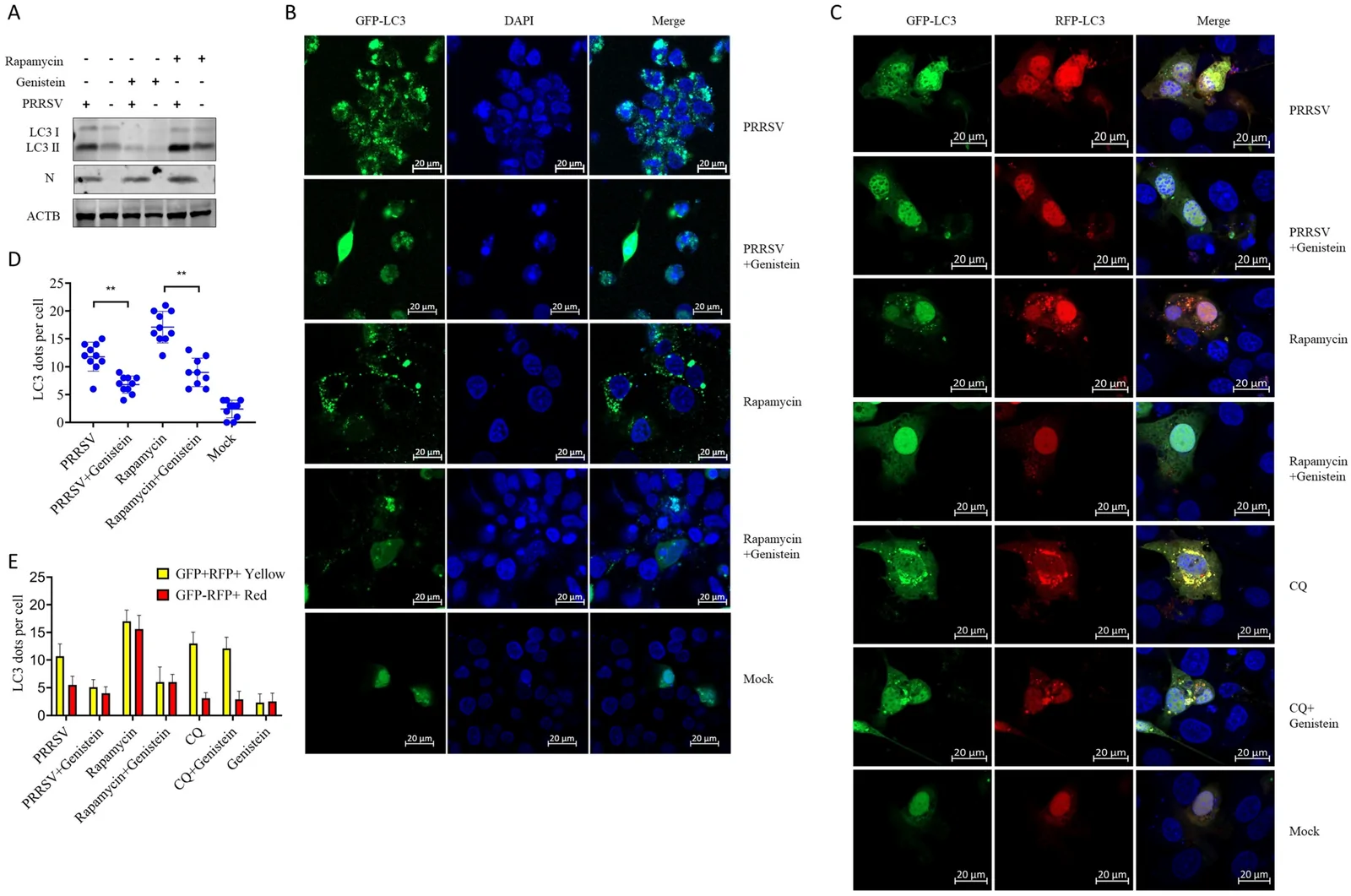
Genistein inhibited autophagy. (**A**) Marc-145 cells were treated with rapamycin or infected with PRRSV, then genistein was added for 6 h, WB was performed to test the level of autophagy marker LC3. (**B**) Marc-145 cells were transfected with GFP-LC3 plasmids for 24 h, following treatment with rapamycin or infection with PRRSV then addition of genistein for 6 h; GFP-LC3 punctas were observed with confocal microscope. (**C**) Marc-145 cells were transfected with RFP-GFP-LC3 plasmids for 24 h, following treatment with rapamycin, CQ, or infection with PRRSV, then addition of genistein for 6 h; RFP-GFP-LC3 punctas were observed using confocal microscope. (**D**) The numbers of LC3 punctas in GFP positive cells were quantitatively analyzed. (**E**) The numbers of red and yellow punctas of ten fields were quantitatively analyzed. The data are presented as the mean of three independent experiments. **, *p* < 0.01.

It has been reported that PRRSV infection induces autophagy to promote viral replication [23,24,25]. To determine whether cellular autophagy levels changed following genistein treatment during PRRSV infection, we investigated the expression of LC3-I and LC3-II by WB. As shown in Figure 6A, genistein treatment decreased the LC3-II level in both mock- and PRRSV-infected cells significantly, suggesting that genistein attenuates autophagy in PRRSV-infected cells. We further investigated the formation of characteristic LC3 puncta in cells transfected with GFP-LC3 plasmids. GFP-LC3 exhibited a perinuclear punctate distribution pattern in PRRSV-infected cells, which was diminished following genistein treatment (Figure 6B). In rapamycin-treated Marc-145 cells, GFP-LC3 proteins were distributed as discrete punctate foci, and genistein treatment similarly attenuated the rapamycin-induced punctate pattern. Quantitative analysis of LC3 puncta numbers further confirmed that genistein treatment attenuates autophagy levels in PRRSV-infected cells. In order to investigate the mechanism of how genistein influences autophagy, a tandem reporter construct RFP-GFP-LC3 was used. The GFP moiety of this tandem autophagosome marker is sensitive to lysosomal proteolysis and quenching in acidic pH, while the RFP is not influenced. And therefore, the green fluorescent component of the composite yellow fluorescence for this RFP-GFP-LC3 reporter is lost after autophagosome fusion with lysosomes, the dots presented as red in autolysosomes. While, the yellow dots are the principal form of expression in autophagosomes. We transfected the cells with RFP-GFP-LC3, following infection with PRRSV or treatment with genistein, rapamycin, or CQ. The results showed that there were few yellow autophagosomes and red autolysosomes in genistein-treated cells, suggesting that genistein could not induce autophagy and autophagic flux; in CQ-treated cells and PRRSV-infected cells, the increased yellow autophagosomes suggest that CQ and PRRSV infection inhibited autophagic flux. In rapamycin-treated cells, abundant yellow autophagosomes and red autolysosomes were observed at the same time, indicating that rapamycin induced autophagosome formation and complete autophagic flux. However, upon addition of genistein to rapamycin-treated and PRRSV-infected cells, both yellow autophagosomes and red autolysosomes were reduced, suggesting genistein inhibited the formation of autophagosomes. Upon addition of genistein to CQ-treated cells, the number of yellow autophagosomes did not reduce, suggesting that genistein did not promote autophagic flux (Figure 6C,E). All these results suggest that genistein inhibits autophagosome formation at the initial stage rather than influences autophagic flux. Therefore, PRRSV may exhibit reduced replication in cells with genistein-induced autophagosome reduction.

## 3. Discussion

Genistein, as an isoflavone derived from soybeans and soy feed products, was tested for its antiviral effect as a dietary supplementation in vivo [20,21]. In this study, we investigate the antiviral effect of genistein in vitro and the molecular mechanisms. Our findings indicate that genistein significantly decreased the replication of PRRSV in Marc-145 cells as viral protein expression, RNA levels, and viral titers of the progeny virus, and its antiviral mechanism was efficacious against diverse PRRSV strains. Our results revealed that genistein inhibited PRRSV infection in Marc-145 cells at multiple stages of the PRRSV replication cycle, including adsorption, entry, and replication, without affecting viral assembly or release. These findings support the concept of incorporating high-isoflavone soybean cultivars—some of which can achieve total isoflavones concentrations approaching 4500 mg/kg—into swine feed formulations as a practical, cost-effective strategy for PRRSV prevention.

The SI is a critical metric for evaluating the clinical utility of antiviral candidates. In this study, genistein exhibited a CC_50_ of 344.01 μM and an EC_50_ of 21.53 μM in Marc-145 cells, yielding an SI of approximately 16. This value meets the minimum acceptable threshold for antiviral candidates (SI ≥ 10); it is moderately low compared to the stringent benchmarks typically applied to purpose-designed synthetic antiviral drugs. It could be interpreted within the unique context of genistein as a dietary isoflavone. Notably, Greiner et al. demonstrated that dietary supplementation with genistein effectively reduced PRRSV viremia and improved growth performance in experimentally infected pigs without overt toxicity, indicating that therapeutic concentrations are readily achievable in vivo. Collectively, genistein as a feed ingredient, supports its continued evaluation as a practical dietary adjunct for PRRSV control in swine production. Previous studies have reported that PRRSV entry into Marc-145 cells requires the Src-EGFR-PI3K-AKT signaling axis [22]. PRRSV infection rapidly activates EGFR phosphorylation within 30 min, followed by downstream activation of PI3K and AKT. As an inhibitor of tyrosine kinase, it has been reported that genistein could inhibit Src family kinases and attenuates EGFR phosphorylation in several cells [8,26,27]. Therefore, genistein inhibits PRRSV entry possibly through its broad tyrosine kinase inhibitor activity. Whether genistein exerts its antiviral effect through its kinase inhibition function and the accurate target site both need to be verified in the future.

Genistein exhibits a dual role in modulating autophagy depending on the cells and tissue type. In vascular smooth muscle cells (VSMCs), genistein activates autophagy via the LKB1-AMPK signaling pathway. Similarly, genistein-induced autophagy has been implicated as a potential therapeutic strategy for neurodegenerative diseases [28,29]. Conversely, genistein suppresses autophagy by regulating the p62/RANKL axis to alleviating osteoporosis [30,31]. These findings suggest that genistein can either induce or inhibit autophagy in a cell type-specific manner. PRRSV infection activated autophagy in Marc-145 cells, which is thought to be beneficial for viral replication. In this study, we observed that genistein reduced rapamycin treatment and PRRSV infection caused autophagy in Marc-145 cells, and genistein treatment decreased LC3-II level and GFP-LC3 punctas. In order to investigate the mechanism of how genistein influences autophagy, RFP-GFP-LC3 was used to indicate the turn of autophagosomes to red autolysosomes. The results showed that genistein mainly inhibits autophagosome formation at the initial stage rather than influences autophagic flux. PRRSV is known to induce incomplete autophagy and arrest autophagosome–lysosome fusion to establish replication platforms [24]. These results suggest that the inhibition of virus infection-induced autophagy by genistein is associated with its antiviral activity.

In conclusion, our study demonstrates that genistein inhibits PRRSV infection at several stages of the PRRSV replication cycle, with the most prominent effects occurring at the entry and replication phases.

These findings provide a mechanistic basis for the previously observed reductions in viremia and improvements in growth performance in PRRSV-infected pigs supplemented with genistein. Given its natural origin, established safety profile, and dual functionality as an antiviral and growth-promoting agent, genistein represents a promising therapeutic candidate for PRRSV control.

## 4. Materials and Methods

### 4.1. Cells, Viruses, and Drugs

Marc-145 cells used in this study were cultured in Dulbecco’s Modified Eagle’s medium (DMEM; Sigma-Aldrich, St. Louis, MO, USA) supplemented with 10% fetal bovine serum (Gibco, Grand Island, NY, USA) at 37 °C in 5% CO_2_. Primary PAMs were preserved in our laboratory, and cultured in RPMI 1640 medium (Gibco, USA) containing 10% FBS at 37 °C in 5% CO_2_. PRRSV strains (VR2332, HLJWK108, and LNTZJ1341) were propagated on Marc-145 cells, as previously described. Genistein was purchased from sigma (Shanghai, China) at 98% purity, dissolved in dimethylsulfoxide (DMSO) at a stock concentration of 100 mM, aliquoted into small volumes to avoid repeated freeze–thaw cycles, and stored at −20 °C. Prior to each experiment, the stock solution was freshly thawed and diluted into cell culture medium to achieve the desired working concentration, ensuring that the final DMSO concentration did not exceed 0.1% (*v*/*v*). Genistein concentration at the 0 μM treatment group contained 0.1% DMSO.

### 4.2. Antibodies

The antibodies for PRRSV N were generated by immunization of mice with purified N protein and stored in our laboratory. Antibody against β-Actin was procured from Affinity Bioscience (T0022, Beijing, China). FITC-conjugated goat anti-pig IgG antibody was purchased from Sigma-Aldrich (St. Louis, MO, USA). DAPI stains were purchased from Beyotime Biotechnology (C1006, Shanghai, China).

### 4.3. Cells Viability Assay

Cell viability was determined by using Enhanced Cell Counting Kit-8 (CCK-8) assays, according to the manufacturer’s instructions (Beyotime Biotechnology, Shanghai, China). Marc-145 cells were seeded into 96-well plates and cultured for 12 hpi. The medium was changed to DMEM supplemented with genistein at different concentrations (0 μM, 20 μM, 40 μM, and 80 μM) for 36 h. Then, 10 µL CCK-8 working solution was introduced into each well, and the plates were maintained at 37 °C for 2 h. The absorbance at 450 nm was detected with a microplate reader (Bio-Rad, Hercules, CA, USA). Relative cell viability was expressed as a percentage of the values obtained from vehicle-treated control cells.

### 4.4. Virus Titration

Marc-145 cells were seeded in 96-well plates in DMEM–8% FBS and cultured for 24 h. Then, confluent monolayers were challenged with 100 µL of virus with ten-fold serial dilutions in serum-free DMEM at 37 °C for 1 h. Eight replicates were set up per dilution. Unbound virus was removed by three PBS washes at 2 hpi, and cells were maintained in DMEM–2% FBS. The cytopathic effect (CPE) development was scored at 5 dpi, and viral titers were expressed as TCID_50_/mL calculated according to Reed and Muench.

### 4.5. Reverse Transcription-qPCR (RT-qPCR)

Total RNA was extracted using an RNA extraction kit (Tiangen Biotech, Beijing, China) according to the instruction manual. RT-qPCR was performed to quantify the copies of PRRSV RNA. The One Step PrimeScript™ RT-PCR Kit (TaKara, Dalian, China) was used according to the manufacturer’s instructions. The RT-qPCR was performed with the following procedures: reverse transcription at 42 °C for 6 min, initial denaturation at 95 °C for 10 min, followed by denaturation at 95 °C for 10 s annealing and extension at 60 °C for 30 s with 40 cycles. Actin gene was used as the internal reference control in the same sample. The primers and probe sequences were used: N forward primer: 5′-TTGTGTCTGTCGTCGATCCAG-3′, reverse primer: 5′-AAACTCCACAGTGTAACTTATCCTC-3′ and N probe: 5′-CGCTGGAACTTGTGCCCTGTCA-3′. Actin forward primer: 5′-TGACTGACTACCTCATGAAGATCC-3′, reverse primer: 5′-TCTCCTTAATGTCACGCACGATT-3′, and Actin probe: 5′-CGGCTACAGCTTCACCACCACGGC-3′.

### 4.6. Western Blot

Marc-145 cells were harvested by centrifuge at 3000 rpm and lysed by adding RAPI buffer. Cell lysis supernatant was collected and mixed with SDS-PAGE loading buffer. The samples separated on 12% SDS-PAGE and the protein was transferred onto polyvinylidene difluoride (PVDF) membrane. Membranes were probed with primary antibodies: ACTB mAb (1:5000) and mAb against PRRSV N protein for 1 h. Then, the membrane was washed thrice with PBST and incubated with DyLight 800-labeled goat anti-mouse IgG (1:10,000) in PBS for 2 h at room temperature.

### 4.7. Time-of-Addition

The inhibitory kinetics of genistein on the PRRSV life cycle was performed according to time-of-addition assay. Marc-145 cells were subjected to genistein treatment at three distinct temporal intervals during PRRSV infection: pre-treatment, co-treatment, or post-treatment. For pre-treatment, the genistein was added to cells at 2 h prior to infection; following genistein incubation, the compound was removed by PBS washing, and cells were subsequently inoculated with PRRSV for 2 h. For co-treatment, cells were treated simultaneously with PRRSV and genistein at 37 °C for 2 h, after removing genistein and the unbound virus with PBS washing, and fresh maintenance media were added. For post-treatment, the cells were first infected with PRRSV at 37 °C for 2 h and subsequently washed with PBS prior to genistein administration. Virus titer and RNA levels were determined by TCID_50_ assay and RT-qPCR at 24 hpi, respectively.

### 4.8. Attachment, Penetration, Replication, Assembly and Release Assays

In the attachment assay, Marc-145 cells in a 12-well cell culture plate were incubated with PRRSV (MOI = 0.1) and genistein at 4 °C for 1 h. The unbound virus was removed by PBS washing and cultured with maintenance medium at 37 °C for 24 h. Then, RT-qPCR was performed to quantify the copy number of PRRSV RNA.

In the penetration assay, Marc-145 cells were incubated with PRRSV (MOI = 0.1) at 4 °C for 1 h to allow virus adsorption. The unbound virus was removed by washing with cold PBS, then the cells were incubated with genistein at 37 °C for 1 h. After incubation, the cells were washed with citric acid (40 mM citric acid, 10 mM KCl, 135 mM NaCl, pH 3.0) and with PBS three times. Then, Marc-145 cells were cultured with maintenance medium at 37 °C for 24 h. RT-qPCR was performed to quantify the copy number of PRRSV RNA.

In the viral replication assay, Marc-145 cells were incubated with PRRSV (MOI = 0.1) at 37 °C for 1 h, then washed with PBS. The infected cells were incubated with genistein at 37 °C. RT-qPCR was performed to quantify the copy number of PRRSV RNA at 6 hpi.

In the viral assembly and release assay, Marc-145 cells were incubated with PRRSV (MOI = 0.1) at 37 °C for 1 h and culture for 20 h, following by washing with PBS, genistein was added in maintenance DMEM medium and cultured for another 6 h at 37 °C. Then the cells and supernatants were respectively collected to quantify the viral RNA by RT-qPCR. The cells and supernatants were collected, and virus titers were tested by TCID_50_ assay.

### 4.9. The Level of Autophagy Assay

Marc-145 cells were treated with rapamycin or infected with PRRSV for 12 h, then genistein was added to cells for 6 h. WB was performed to test the protein level of LC3. For the detection of autophagosomes, the cells were first transfected with GFP-LC3 for 24 h, following treatment with rapamycin or infection with PRRSV, then the addition of genistein for 6 h. The cells were fixed, and stained with 4′-6-diamidino-2-phenylindole (DAPI, Invitrogen, Carlsbad, CA, USA). GFP-LC3 punctas were observed with confocal microscope (LSM980-ZEISS, Oberkochen, Germany). The numbers of LC3 punctas in GFP-positive cells were quantitatively analyzed.

### 4.10. Statistical Analysis

All experiments in the study were performed with three independent replicates, and the error bars indicate the standard deviation (SD). Statistically significant differences were analyzed using the Student’s *t* test for comparisons between two groups and one-way analysis of variance (ANOVA) with the Tukey multiple-comparison test using Graphpad Prism 8.0 software. *, *p* < 0.05 was considered to indicate statistical significance; **, *p* < 0.01; ***, *p* < 0.001; ns, not significant.

## Data Availability

The original contributions presented in this study are included in the article. Further inquiries can be directed to the corresponding authors.
